# PKM2 regulates Gli1 expression in hepatocellular carcinoma

**DOI:** 10.3892/ol.2014.2441

**Published:** 2014-08-12

**Authors:** QIURAN XU, XIN LIU, XIN ZHENG, YINGMIN YAO, QINGGUANG LIU

**Affiliations:** 1 Department of Hepatobiliary Surgery, The First Affiliated Hospital of Medical College, Xi’an Jiaotong University, Xi’an, Shaanxi 710061, P.R. China; 2 Emergency Department, Zhejiang Provincial People’s Hospital, Hangzhou, Zhejiang 310014, P.R. China; 3 Department of Neurosurgery, The First Affiliated Hospital of Medical College, Xi’an Jiaotong University, Xi’an, Shaanxi 710061, P.R. China

**Keywords:** hepatocellular carcinoma, PKM2, Gli1

## Abstract

Hedgehog (Hh) signaling and the pyruvate kinase isoenzyme M2 (PKM2 or M2-PK) are often involved in tumorigenesis and growth. Aberrant activation of Hh signaling is found in a variety of malignancies. In tumor cells, PKM2 determines whether glucose is used for the synthesis of cellular building blocks or the production of lactate for energy regeneration; it associated with the Warburg effect. Gli1 is a downstream molecule of the Hh signaling pathway; however, the association between Hh signaling and PKM2 is not well understood. In the present study, it was identified that PKM2 and Gli1 expression levels were significantly elevated in hepatocellular carcinoma (HCC) compared with para-carcinoma. *In vitro* study revealed that overexpression of PKM2 in HepG2 cells upregulated the transcription of Gli1, while the ablation of PKM2 by shRNA caused the downregulation of Gli1 gene expression. Gli1 transcription could be rescued by PKM2. Overall, these findings suggest that PKM2 is a regulator of Gli1 gene expression in HCC, and may contribute to tumorigenesis through Gli1.

## Introduction

Hepatocellular carcinoma (HCC) is the most common form of liver cancer and the third leading cause of cancer-related mortality worldwide (1–3). Its incidence has increased in recent years. The etiology of HCC includes alcohol abuse, chronic viral hepatitis, environmental carcinogens or genetic disorders. Although several risk factors for HCC development are known, the therapeutic options for this disease are very limited. Hepatic resection remains the most effective treatment (4), but the prognosis of HCC is generally poor, with high postoperative recurrence and invasiveness of primary tumor responses. Therefore, it is important to explore novel molecular targets for treatment strategies that have the potential to significantly improve the prognosis of HCC.

The role of Hedgehog (Hh) signaling in human cancer has been established through the studies of basal cell nevus syndrome (5), a rare hereditary disorder with a high risk of basal cell carcinomas. The activation of Hh signaling has been observed in numerous types of cancer, such as prostate cancer, gastrointestinal cancer and HCC (6–8). The Hh signaling pathway is a highly conserved system, which plays a crucial role in cell differentiation, proliferation and tissue patterning (9). In vertebrate organisms, the signaling pathway is initiated by the ligands (Desert, Indian and Sonic hedgehog) that bind to the membranous receptor patched (Ptch). Ptch alleviates the suppression on smoothened (Smo) that triggers a series of intracellular events by activating glioma-associated oncogenes (Gli1, Gli2 and Gli3) that induce the expression of numerous target genes and regulate differentiation, proliferation and extracellular matrix interactions (9–11). Gli1 is one of downstream effectors of Hh signaling and acts as a transcription factor to promote cell growth and inhibition of apoptosis (12,13). Gli1 is overexpressed in several cancer tissues including glioblastoma (14), breast cancer (15) and pancreatic adenocarcinoma (16). Moreover, the transcription of Hh signaling-associated molecules (such as Shh, Smo and Gli1) is also overexpressed in some cases of HCC (17,18).

Numerous types of cancer cells require increased glucose uptake with a concomitant decrease in oxidative phosphorylation, even in the presence of oxygen. This phenomenon of aerobic glycolysis with increased lactate production has been named as the Warburg effect (19). A previous study demonstrated that expression of pyruvate kinase M2 (PKM2 or M2-PK) is a key event in determining this metabolic phenotype, and tumor expression of M2 provides a proliferative advantage *in vitro* and *in vivo* (20). Pyruvate Kinase (PK) is a key regulatory enzyme in glycolysis and it has four known isoforms, including L, R, M1 and M2. PKM2 plays a central role in the metabolism of cancer cells and is expressed in a broad range of human cancers. PKM2 can directly regulate gene transcription, which may occur in both active tetrameric and inactive dimeric forms (21–23). In addition, certain tyrosine kinases may also be responsible for the Warburg effect in cancer, as they can phosphorylate glycolytic enzymes, including PKM2, and then promote tumor growth (24).

However, the exact role of PKM2 in tumor growth and maintenance is not clear. Moreover, there is currently no study showing a correlation between PKM2 and Hh signaling. The present study identified a novel regulatory mechanism for PKM2, as a regulator for Gli1 expression in HCC.

## Materials and methods

### Cell lines and reagents

HepG2 cells were purchased from the ATCC (Manassas, VA, USA) and L-O2, Huh-7 and Hep3B cells were purchased from the National platfom of Experimental Cell Resources for Sci-Tech (Beijing, China) and maintained in Dulbecco’s modified Eagle’s medium (DMEM; Hyclone, Logan, UT, USA) with 10% (v/v) fetal bovine serum (FBS; Gibco-BRL, Carlsbad, CA, USA). 293T cells were purchased from the cell bank and maintained in RPMI-1640 (Hyclone) with 10% (v/v) FBS. The cells were incubated at 37°C in a 5% CO_2_ humidified atmosphere for 24 or 48 h The pS-FLAG-SBP (SBP) vector was provided by Dr Xin Zheng (The First Affiliated Hospital of Xi’an Jiaotong University, Xi’an, China), and the pcDNA3-Gli1 human Gli1 expression vector and pIRES2-S-SBP-FLAG plasmid were provided by Dr Xin Zheng. Vector PLKO was purchased from Addgene (Cambridge, MA, USA), it is a replication-incompetent lentiviral vector for the expression of shRNAs. Rabbit polyclonal anti-human PKM2 and mouse monoclonal anti-human ACTB antibodies were purchased from Cell signaling Technology, Inc. (Beverly, MA, USA), while rabbit polyclonal anti-human Gli1 and mouse monoclonal anti-human HA antibodies were purchased from Santa Cruz Biotechnology, Inc. (Santa Cruz, CA, USA). GFP-PKM2 was obtained from OriGene Technologies, Inc., tagge with green fluorescent protein. Patients provided written informed consent.

### Patients and tissue samples

A total of 63 patients at the The First Affiliated Hospital of Xi’an Jiaotong University with HCC were enrolled in the study between January 2009 and October 2009, including 49 males and 14 females (mean age, 52 years; range, 35–71 years) who had not received pre-operative chemotherapy or embolization. Following routine X-ray, abdominal ultrasonography and computed tomography, all patients underwent liver resection, including curative resection for early HCC and palliative resection for advanced HCC. Tumor tissue and matched adjacent normal tissue specimens (>2 cm distant from the resection margin) were collected and immediately stored in liquid nitrogen for quantitative polymerase chain reaction (qPCR) and paraformaldehyde for immunohistochemistry, respectively. Clinical data were obtained from the patients’ medical records. Subsequently, histopathological Edmonson classification, clinical tumor-node-metastasis (TNM) grading, maximum tumor diameter and the normal tumor-adjacent tissues were all confirmed by an experienced pathologist who was blinded to the clinical information.

Written informed consent was obtained from all patients. The ethics committee of Xi’an Jiaotong University (Xi’an, China) approved all protocols according to the 1975 Declaration of Helsinki.

### Immunohistochemistry

Immunohistochemistry was performed on paraformaldehyde-fixed paraffin sections. The sections were dewaxed and dehydrated. Following rehydration, endogenous peroxidase activity was blocked for 30 min using a methanol solution containing 0.3% hydrogen peroxide. After antigen retrieval in citrate buffer, the sections were blocked overnight at 4°C, and then separately incubated with the primary antibodies directed against Gli1 and PKM2, at 4°C overnight. The primary antibody was detected using biotinylated polyclonal goat anti-mouse IgG (H+L) and polyclonal goat anti-rabbit IgG (H+L) secondary antibodies (Zhongshan Golden Bridge Biotechnology Co., Ltd., Beijing, China) according to the manufacturer’s recommendations. The staining of the sections was performed using the avidin-biotin-peroxidase complex (Zhongshan Golden Bridge Biotechnology Co., Ltd.) for Gli1 and PKM2. The sections were visualized with diaminobenzidine and counterstained with hematoxylin, then dehydrated in alcohol and xylene and mounted onto glass slides.

All sections were assessed independently by two experienced pathologists. The staining results for the two proteins (Gli1 and PKM2) were semi-quantitatively expressed by an immunohistochemical score combined with the percentage of tumor cells showing specific immunoreactivity. Staining intensity was scored as follows: 0, none; 1, weak; 2, moderate; and 3, strong. The percentage of positive carcinoma cells was scored as follows: 0, <5%; 1, 6–25%; 2, 26–50%; 3, 51–75%; and 4, >75%. The staining intensity and average percentage of positive tumor cells were assayed for 10 independent high-magnification (x400) fields (Olympus CX21; Olympus Corporation, Tokyo, Japan). The total score was calculated by multiplying the staining intensity score by the percentage of positive tumor cells score. Sections with a total score of >1 were defined as exhibiting positive staining for the above two proteins.

### Cell lysis, immunoprecipitation and western blotting

293T cell transfections, protein extract preparations, immunoprecipitation and western blot analysis were performed as previously described (25,26). Briefly, for immunoprecipitation, cells were lysed with ice-cold NETN100 buffer [20 mM Tris-HCl, pH 8.0 (Sangon Biotech Co., Ltd., Shanghai, China), 100 mM NaCl (Sangon Biotech Co., Ltd.), 1 mM EDTA (Sangon Biotech Co., Ltd.), 0.5% Nonidet P-40 (Amresco, Solon, OH, USA)] containing 10m M NaF and 50 mM b-glycerophosphate, and then subjected to sonication for 12 sec. Supernatants were incubated with indicated antibodies (anti-PKM2, -Gli1, -ACTB and -HA) and G-protein-conjugated sepharose beads (Amersham Pharmacia Biotech, Inc., Piscataway, NJ, USA). Precipitates were washed three times with NETN100, and then subjected to SDS-PAGE and western blotting with the indicated antibodies. To examine the PKM2 expression, cell pellets were lysed with 400 ml NETN100 buffer. Following centrifugation at 13,000 × g for 20 min, the supernatants were termed 100 mM NaCl samples. The insoluble pellets were collected, washed with ice-cold phosphate-buffered saline (PBS) and incubated with 400 ml NETN300 buffer (20 mM Tris-HCl, pH 8.0, 300 mM NaCl, 1 mM EDTA, 0.5% Nonidet P-40) on ice. After centrifugation, the supernatants were termed 300 mM NaCl samples. The remaining pellets were washed twice with ice-cold PBS and then treated with 200 ml 0.2 N HCl. The supernatants were neutralized with 40 ml 1 N NaOH, and termed 0.2 N HCl fractions. Each fraction sample was loaded onto 7.5% SDS-PAGE gels for western blotting with the indicated antibodies. Western blotting was quantified using Quantity One version 4.6.2 (Bio-Rad Laboratories, Philadelphia, PA, USA).

### RNA isolation and qPCR

The expression of Gli1 in HepG2 cells was determined by reverse transcription of total RNA, followed by qPCR analysis. Total RNA (1 μg) was reverse-transcribed with random hexamers using Superscript II reverse transcriptase (Invitrogen Life Technologies, Carlsbad, CA, USA) according to the manufacturer’s instructions. qPCR was performed on a Bio-Rad iCycler using iQ™ SYBR Green (Bio-Rad Laboratories, Inc., Hercules, CA, USA) with the following primers: Forward, 5′-GAAGGTGAAGGTCGGAGT-3′ and reverse, 5′-GTCCAGGCTGGCATCCGACA-3′ for Gli1; forward, 5′-GAAGGTGAAGGTCGGAGT-3′ and reverse, 5′-GAAGATGGTGATGGGATTTC-3′ for GAPDH; forward, 5′-GGCAGAGGCTGCCATtTAtCAtTTaCAgTTgTTcGAGGAACTCCGCCGCCT-3′ and reverse 5′-AGGCGGCGAGTTCCTCGAACAACTGTAAATGAT AAATGGCAGCCTCTGCC-3′ for PKM2 shRNA-resistant 1408; and forward, 5′-AGAGGCTGCCATCTAtCAtTTaCAgTTgTTcGAaGAACTCCGCCGCCTGGC-3′ and reverse, 5′-GCCAGGCGGCGGAGTTCTTCGAACAACTGTAAAT GATAGATGGCAGCCTCT-3′ for PKM2 shRNA-resistant 1411. PKM2 shRNA resistant 1408 and PKM2 shRNA resistant 1411 were used to generate the PKM2 shRNA resistant 1408 and PKM2 shRNA resistant 1411 plasmids (QuikChange Site Directed Mutagenesis kit, Stratagene, Agilent Technologies, Inc., Santa Clara, CA, USA).

### Glutathione S-transferase (GST) pull-down assay

293T cells were used for the GST pull-down assay as they exhibit a higher transfection efficiency than the other cell lines. First, to produce 293T cells overexpressing HA-tagged Glil, the 293T cells were grown in DMEM containing 10% FBS. Next, 2×10^6^ cells were seeded in 10-cm dishes 24 h prior to transfection with 5 mg of the HA-Gli1 plasmid using Lipofectamine 2000 reagent (Invitrogen Life Technologies), according to manufacturer’s instructions. Subsequently, 1 mg of GST-PKM2 or GST (OriGene Technologies, Inc., Rockville, MD, USA) as a control was incubated with the cell lysates from the 293T cells overexpressing HA-tagged Gli1. Glutathione beads (Sigma-Aldrich, St. Louis, MO, USA) were then added and incubated for 2 h. The bound proteins were eluted with sample loading buffer and analyzed by immunoblotting with HA antibodies. For endogenous immunoprecipitation, 293T cell lysates were immunoprecipitated with normal mouse immunoglobulin G as a control, followed by incubation with protein A beads (Sigma-Aldrich). The bound proteins were subjected to immunoblot analysis with PKM2 antibody.

### Transfection

293T cells were grown in DMEM containing 10% FBS. A total of 2×10^6^ cells were seeded in 10-cm dishes 24 h prior to transfection. Cells were subsequently transfected with 5 mg GFP-PKM2, HA-Gli1, PKM2 shRNA 1408 or shRNA 1411, and vector PLKO plasmids using Lipofectamine 2000 reagent (Invitrogen Life Technologies), according to the manufacturer’s instructions.

### Statistical analysis

Data are presented as the mean ± standard error of the mean. Mann-Whitney U tests were used for statistical analysis unless otherwise indicated.

## Results

### Gli1 and PKM2 expression in HCC

**Figure 1:**
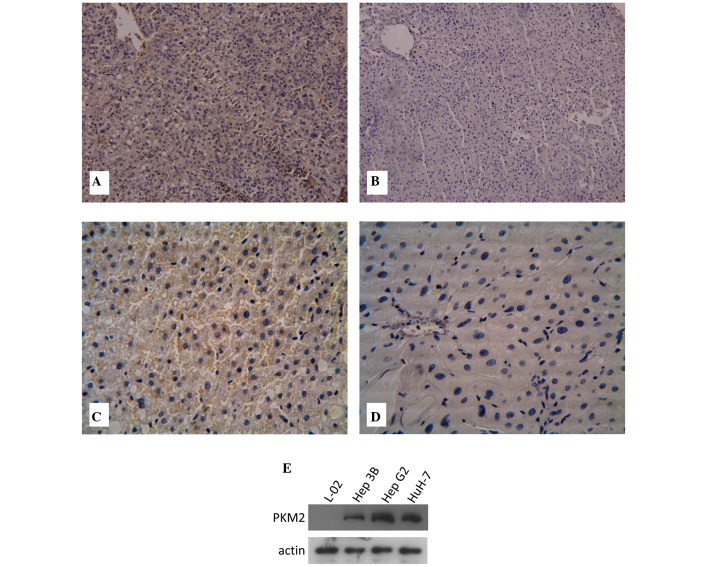
Gli1 and PKM2 expression in HCC. The expression pattern of (A) Gli1 in HCC tumor tissues and (B) Gli1 in adjacent normal tissues and (C) PKM2 protein in HCC tumor tissues and (D) PKM2 protein adjacent normal tissues by immunohistochemistry. Magnification, ×400. (E) Expression of PKM2 in human normal liver L-02 cells and HCC cell lines (Hep3B, HepG2 and HuH-7) by western blot analysis. PKM2, pyruvate kinase isoenzyme M2; HCC, hepatocellular carcinoma.

To examine Gli1 expression in HCC, a total of 63 pairs of HCC and adjacent normal tissues from HCC patients were examined by immunohistochemistry. Gli1 protein levels in HCC and adjacent normal tissues were assessed using a Gli1-specific antibody. It was found that Gli1 protein expression was positive in 57 out of 63 tumor tissues (90.48%), including 17 (26.98%) highly positive (+++) cases. By contrast, Gli1 protein expression was observed in 21 of 63 normal tissues (33.33%) and none of these tissues were highly positive (Fig. 1A and B and Table I). Cox regression analysis showed that there was a significant correlation between Gli1 expression and tumor invasiveness, including histological differentiation, portal vein tumorous thrombogenesis, lymph node invasion and TNM stage (Table II).

The expression of PKM2 protein was examined by immunohistochemistry. As shown in Fig. 1C and D, PKM2 was mainly expressed in the cytoplasm. Immunohistochemistry indicated that the levels of PKM2 in HCC tissues were significantly higher than that in adjacent normal tissues (4.44±1.54 vs. 2.13±1.34; P<0.05). Furthermore, the protein expression levels of PKM2 in three HCC cell lines (Hep3B, HepG2 and HuH-7) and one human normal liver cell line (L-02 cell line) were examined by western blot analysis. It was found that PKM2 was highly expressed in the three HCC cell lines, but not in the L-02 cell line (Fig. 1E).

### Gli1 directly interacts with PKM2

**Figure 2:**
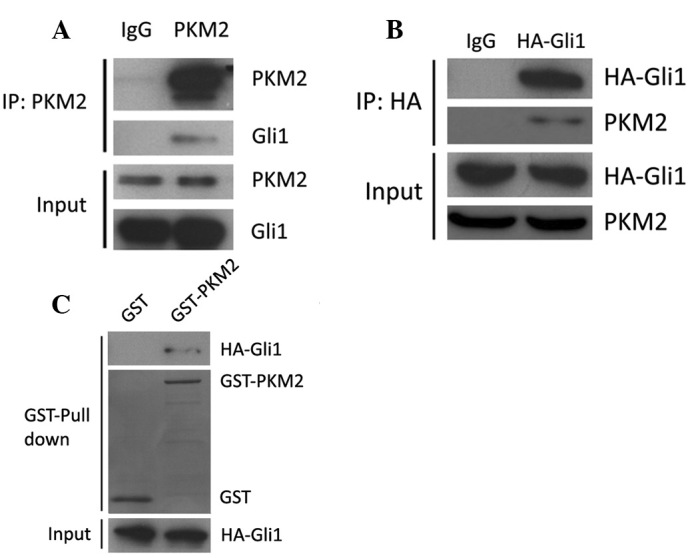
Gli1 directly interacts with PKM2. (A) 293T cell lysates were incubated with PKM2 antibody or normal mouse IgG, and then immunoblotted with PKM2 and Gli1 antibodies. (B) 293T cells were transiently transfected with HA-Gli1. The cell lysates were immunoprecipitated with anti-HA resin, and then immunoblotted with HA and PKM2 antibodies. Irrelevant IgG was used as the immunoprecipitation control. (C) GST-pull down showed a direct interaction between PKM2 and Gli1 *in vitro*. 293T cells were transfected with HA-Gli1. The cell lysates were incubated with GST or GST-PKM2. PKM2, pyruvate kinase isoenzyme M2; IgG, immunoglobulin G; GST, glutathione S-transferase.

In order to investigate the functional association between PKM2 and Gli1, we first examined the physical associations between the two molecules using immunoprecipitation. As shown in Fig. 2A, Gli1 protein was precipitated by specific PKM2 antibody in 293T cell lysates. It was also examined whether PKM2 could be precipitated by Gli1 antibody. Due to the lack of specific Gli1 antibody for immunoprecipitation, 293T cells were transfected with HA-Gli1. The cell lysates were subjected to incubation with HA antibody and then immunoblotted with PKM2 antibody. It was observed that HA-Gli1 could interact with PKM2 (Fig. 2B). To examine the direct interaction between PKM2 and Gli1, GST-PKM2 and HA-Gli1 were purified for the GST pull-down assay. As shown in Fig. 2C, GST-PKM2 interacted with HA-Gli1 in the 293T cells. These results indicate that PKM2 is able to interact with Gli1 *in vitro* and *in vivo*.

### PKM2 regulates Gli1 expression

**Figure 3:**
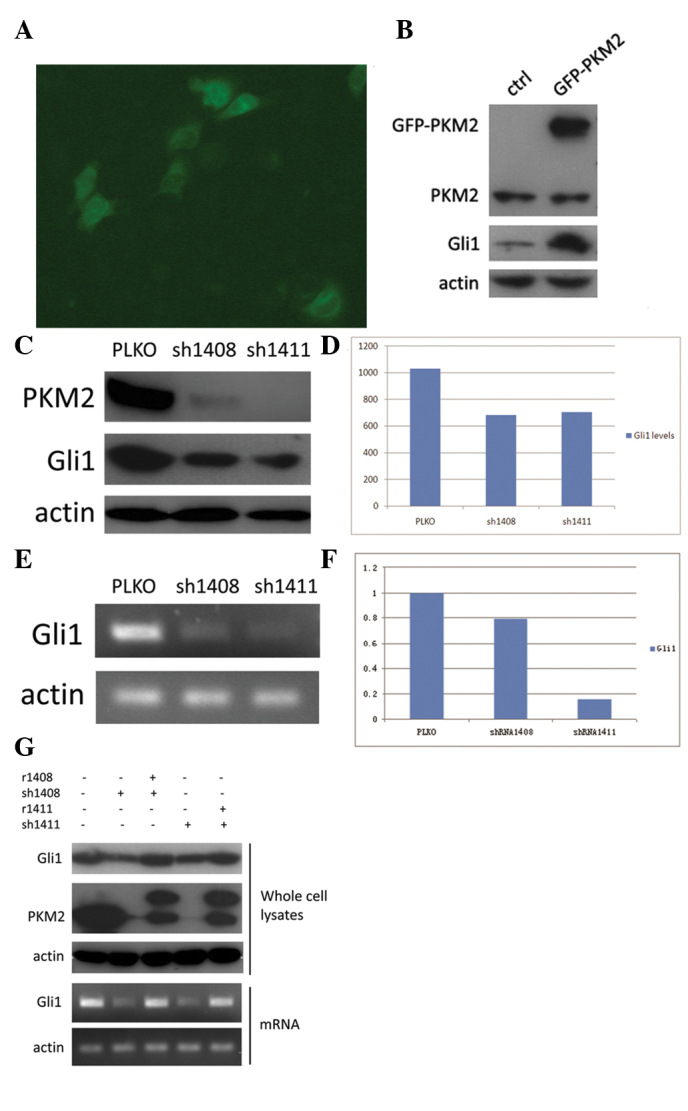
PKM2 regulates Gli1 expression. (A) HepG2 cells were transiently transfected with GFP-PKM2. (B) Overexpression of PKM2 in HepG2 cells increased Gli1 expression. (C and D) PKM2 knockdown by shRNA (1408 and 1411) in HepG2 cells decreased Gli1 expression. PLKO vector was used as a control. (E and F) HepG2 cells were transfected with PKM2 shRNA (1408 or 1411) or PLKO (as a control). After 48 h, quantitative PCR was performed, showing that knockdown of PKM2 downregulated Gli1 at the transcription level. (G) Gli1 expression was analyzed in HepG2 cells by western blotting using whole cell lysates (top) and by reverse transcription PCR using total RNA (bottom) from HepG2 cells. PKM2, pyruvate kinase isoenzyme M2; GFP, green fluorescent protein; PCR, polymerase chain reaction..

To further analyze the association between PKM2 and Gli1, overexpression of GFP-PKM2 was induced in the HepG2 cell line and Gli1 expression was examined (Fig. 3A). It was found that Gli1 expression was significantly upregulated in GFP-PKM2-overexpressing cells compared with normal HepG2 cells (Fig. 3B). As HepG2 cells normally express high levels of PKM2, we also chose to knock down its expression. HepG2 cells were infected with recombinant lentiviruses expressing either PKM2 shRNA 1408 or shRNA 1411, and vector PLKO was used as control. These two distinct ‘targeted’ shRNAs (which were named 1408 and 1411) could significantly ablate PKM2 expression in HepG2 cells (>80% of expression knocked down) (Fig. 3C). Moreover, PKM2 knockdown by shRNA (1408 and 1411) in HepG2 cells markedly decreased the expression of Gli1 mRNA and protein compared with HepG2 cells transfected with the PLKO vector. (Fig. 3C-F).

To further verify the effect of PKM2 shRNAs, which knockdown PKM2 in HepG2 cells, the overexpression of mutated PKM2 resistant to PKM2 shRNA but can express wild-type PKM2 was induced in HepG2 cells. It was found that the reduced Gil1 expression in HepG2 cells with knockdown of PKM2 was completely rescued by reconstituted expression of wild-type PKM2, and these effects were observed at the mRNA and protein levels (Fig. 3G). These results further verify that Gli1 is directly regulated by PKM2.

## Discussion

Early hepatocellular carcinoma (HCC) is rarely diagnosed before the middle or advanced stage (27,28). Recently, certain HCC-associated oncogenes, such as FXR, Plk1, MDR3 and MRP, have been found to be linked with the prognosis of HCC (29–31). However, the exact molecular mechanism of HCC progression is unclear.

Compared with normal cells, cancer cells (including HCC cells) show increased glycolysis and inhibition of oxidative phosphorylation, even in the presence of sufficient oxygen (aerobic glycolysis), which is known as the Warburg effect (19). Aerobic glycolysis is not only conducted to increase the availability of macromolecules for biosynthesis and cell growth, but also to contribute to anti-apoptotic pathways. Increased glucose metabolism protects cells from the pro-apoptotic Bcl-2 family protein, Bim, and attenuates the degradation of the anti-apoptotic protein, Mcl-1 (32). However, some key problems remain unclear, for example, the physiological significance of this glucose-dependent regulation in cancer cells, and the regulatory mechanisms of the Warburg effect remain unknown.

PKM2 has previously been known to be a key enzyme that controls the rate-limiting step of glycolysis and plays a central role in metabolic reprogramming during cancer progression. PKM2 knockdown by siRNA in glioma cells has been demonstrated to induce cell apoptosis and inhibit cell growth, cellular invasion, metabolic activity, ATP levels and glutathione levels (33). Reduced expression of PKM2 protein in lung tumors has been shown to inhibit tumor growth and promote cancer cell apoptosis *in vitro* and *in vivo* (34). Christofk *et al* demonstrated that mice injected with PKM1-overexpressing cells showed a delay in tumor development compared with those injected with PKM2-overexpressing cells (20). Recently, PMK2 was newly characterized as a transcriptional coactivator and protein kinase (21,35), suggesting that PKM2 also has the ability to regulate gene expression, cell cycle progression and metabolism in a feedback loop. All of these findings reflect the important role of PKM2 in tumorigenesis.

The Hh signaling pathway is essential for numerous processes during embryonic development, including cell growth, cell differentiation, patterning and organogenesis (36). However, aberrant Hh signaling is observed in a variety of cancer types. Previous studies have shown that Shh, Gli1, Smo and Patch were overexpressed in HCC, and the Shh signaling pathway played a critical role in HCC tumorigenesis and progression (37,38). But the molecular mechanisms of Hh signaling in HCC remain unclear.

In the present study, we described a previously unknown association between the Hh signaling pathway and PKM2, in which PKM2 affects the Hh signaling pathway by regulating Gli1 transcription levels. By using PCR and immunohistochemistry, it was demonstrated that the levels of Gli1 in HCC tissues were markedly higher than those in adjacent normal tissues. These findings confirmed those of the previous studies showing that Gli1 expression is aberrant in HCC, suggesting that Gli1 may be a key marker for diagnosis (37,38). In the current study, statistical analysis showed there was a significant correlation between Gli1 expression and tumor invasiveness. Additionally, the levels of PKM2 protein in HCC tissues were significantly higher than those in adjacent normal tissues. These data provide a molecular basis for improving the diagnosis and treatment of HCC patients by targeting upregulated PKM2 and Gli1. Furthermore, in the present study, immunoprecipitation and immunoblotting revealed a positive correlation between PKM2 and Gli1. In addition, PKM2 overexpression upregulated Gli1, while knockdown of PKM2 by two different shRNAs caused a significant decrease in Gli1 expression, which could be completely rescued by reconstituted expression of wild-type PKM2. These results suggest that PKM2 may be an important upstream regulator of Gli1 gene expression in HCC.

In summary, the present study has shown that PKM2 is a regulator of Gli1 gene expression in HCC, and PKM2 may contribute to tumorigenesis by controlling Gli1 expression. However, the exact molecular mechanism whereby PKM2 regulates Hh signaling requires further investigation.

## Figures and Tables

**Table I:** Expression of Gli1 protein in the HCC tumor tissues and adjacent normal tissues.

Pathological type	n	Gli1 expression level, n				Positive n (%)	χ^2^	P-value

		−	+	++	+++			
HCC tissues	63	6	8	32	17	57 (90.48)	43.6	<0.05
Adjacent normal tissues	63	42	18	3	0	21 (33.33)		

HCC, hepatocellular carcinoma.

**Table II:** Prognostic factors in Cox proportional-hazards model.

Parameter	RR	95% CI	Wald	P-value
Gender	0.819	0.127–5.274	0.044	0.834
Age	1.033	0.205–5.196	0.002	0.968
HBsAg	0.310	0.057–1.675	1.851	0.174
Cirrhosis	0.237	0.033–1.721	2.027	0.155
Serum AFP	0.530	0.062–4.494	0.339	0.560
Tumor size	0.728	0.181–2.923	0.200	0.655
Differentiation	15.197	2.039–113.291	7.048	0.008^a^
PVTT	6.041	1.395–26.162	5.784	0.016^a^
Lymph node invation	0.032	0.003–0.369	7.627	0.006^a^
Encapsulation	2.484	0.435–14.180	1.048	0.306
Primary tumor	3.105	0.395–24.435	1.159	0.282
TNM stage	75.634	2.757–2.075E3	6.554	0.010^a^
Gli1 mRNA	22.298	2.110–235.510	6.663	0.010^a^

a P<0.05.

RR, relative risk, CI, confidence interval; HBsAg, hepatitis B virus surface antigen; AFP, α-fetoprotein; PVTT, portal vein tumor thrombosis; TNM, tumor-node-metastasis.
